# Design recommendations for studies that evaluate multiple sclerosis fatigue interventions

**DOI:** 10.3389/fpsyt.2023.1287344

**Published:** 2024-01-10

**Authors:** Mahsa Ghajarzadeh, Emmanuelle Waubant, Bardia Nourbakhsh

**Affiliations:** ^1^Department of Neurology, School of Medicine, Johns Hopkins University, Baltimore, MD, United States; ^2^Department of Neurology, University of California, San Francisco, San Francisco, CA, United States

**Keywords:** multiple sclerosis, fatigue, intervention, study design, magnetic resonance imaging

Fatigue, defined as a subjective lack of energy perceived by an individual that interferes with usual or desired activities, is the most common symptom of multiple sclerosis (MS). It is also one of the most disabling symptoms and an important contributor to a lower quality of life among people living with MS (1). People with MS would accept to have more relapses and faster disease progression if they could improve physical and cognitive fatigue (2). Even in people classified as having benign MS according to a low level of disability on the Expanded Disability Status Scale (EDSS) 10 years after onset, close to 80% report having fatigue (3). Despite its prevalence and personal and societal impact, effective, safe, easily accessible, and widely accepted MS fatigue treatments are unavailable.

There are many barriers to researching MS fatigue. First, patients and investigators can refer to different symptoms and concepts under the terminology of fatigue. Increased daytime sleepiness, difficulty with movements, reduced ability to maintain physical or cognitive performance (fatigability), malaise, and dysphoria may be referred to as fatigue (4). Even what is interpreted as “energy” by an individual is a vague concept and is difficult to measure.

Fatigue is a subjective symptom, and none of the objective measures (such as physical activity) would be a great measure of fatigue. While subjective lack of energy may result in less physical activity, many other factors, such as the severity of neurological disability and mood, can affect the level of physical activity more than fatigue. In other words, fatigue would only explain a small proportion of variation in physical activity. At this point, the only way to measure fatigue in a valid way is to ask someone about their perception of lack of energy.

Countless questionnaires have been developed over the years to measure MS fatigue. Most have a look-back period and rely on the person's recall. Recently, the validity of MS fatigue questionnaires has been questioned (5). In other words, it is not quite clear if these questionnaires measure what they are developed to measure (i.e., fatigue). Also, the validated questionnaires currently in use ask patients to evaluate previous fatigue retrospectively, and most have a look-back period of seven to 28 days (6). However, the scores usually do not portray the average fatigue severity in the look-back period and are mostly influenced by the most recent and severe fatigue states (7). The ubiquitous availability of smartphones and their versatility provide a unique opportunity to frequently obtain self-reported and fatigue measures in patients' real-life settings. The methodology, which is referred to as ecological momentary assessment (EMA), involves the repeated sampling of subjects' experiences and behavior in subjects' natural environment and in real-time (8). Using EMA principles in clinical research may reduce measurement bias and improve the chances of finding effective therapies for MS fatigue.

Aside from the difficulty in defining and measuring fatigue, no single underlying cause or pathophysiological model has been discovered for MS fatigue. Alteration in both structural and functional magnetic resonance imaging (MRI) markers has been associated with the presence, severity, and persistence of MS fatigue. For example, changes in fractional anisotropy and mean diffusivity between deep gray matter structures, and lower resting state effective connectivity between prefrontal and caudate nucleus were predictors of fatigue in people with progressive MS (9). As compared to patients who never had fatigue during a longitudinal follow-up, patients with persistent fatigue had higher T2 lesion volume on the MRI (10). Overall, MS fatigue has a multifactorial etiology, and more than 30 factors have been associated with this symptom (11). So, intervening in or fixing one of the associated factors is unlikely to produce a significant change in fatigue in a large proportion of patients.

Despite the aforementioned problems, there is no shortage of studies that have evaluated the effectiveness of various interventions on MS fatigue as their primary or one of their non-primary outcomes. As with any symptomatic treatment trial, changes in fatigue can be seen quickly after starting an intervention, so studies can be short and do not require expensive or sophisticated tools; thus, studying MS fatigue treatment is relatively inexpensive (compared to trials evaluating treatments for MS relapses or disability progression). Despite the abundance of studies investigating various modalities and interventions for MS fatigue, it remains a common and disabling problem among people visiting neurology and MS clinics.

Here, we argue that the design of the studies aimed at evaluating the efficacy of an intervention for MS fatigue is an important consideration. A review of the literature shows that MS fatigue, similar to depression, pain, and migraine headaches (12) is a condition that responds quite well to a placebo, at least on the short term. Comparing the results of open-label studies with masked placebo-controlled trials of an intervention, such as extended-release 4-aminopyridine (fampridine), shows the placebo-responsive nature of MS fatigue. Among 18 open-label studies reporting on the effects of fampridine on MS fatigue, 17 reported an improvement in fatigue after starting the medication. However, of eight blinded, randomized, placebo-controlled trials of fampridine, only 2 reported that fampridine was better than placebo in improving MS fatigue [submitted for publication]. A large, two-center crossover double-blind trial of three medications used to treat fatigue in clinical practice showed that none outperformed placebo (13). In another large double-blind, parallel-group clinical trial, fatigue improvement with modafinil was not superior to the placebo group (14). Even in a placebo-controlled crossover trial of amantadine for MS fatigue, the authors noted the presence of an “important placebo effect” (15).

There are several causes of placebo responsiveness in any clinical trial. One is the placebo effect of the intervention itself. An intervention, by virtue of causing expectancy, may result in improving the medical condition. The second reason for a placebo response is the interactions between the participants and the study personnel, which can improve the studied condition independent of the main intervention(s). Other contributing factors to the placebo response include regression-to-the-mean and measurement error (16).

The magnitude of the placebo response depends on the study design in addition to the condition under study. The placebo response is more pronounced when a smaller proportion of participants are assigned to the placebo group (17). For example, the placebo effect is greater if participants are 2:1 assigned to an active medication compared to a 1:1 assignment. In a study with two or several active comparators (and no placebo), the proportion of observed response that is attributable to placebo response is the largest. The number of study visits or contacts with study personnel can also affect the magnitude of the placebo response, with more frequent contacts resulting in a higher placebo response. Thus, it is not possible to compare the magnitude of a treatment effect observed from an intervention in an open-label, non-placebo-controlled trial with the placebo response reported in a placebo-controlled study. There is no “usual” or maximum placebo response for an intervention or a medical condition.

We want to emphasize that the placebo responsiveness of a condition does not mean that the medical issue is not “real,” psychosomatic, less biological, non-disabling, or not worthy of investigation and treatment. The placebo effect shows that the expectation of receiving benefit from an intervention modulates the neurobiological pathways underlying that symptom or syndrome.

As we mentioned earlier, many MS studies, including those with open-label designs, report on the effectiveness in improving fatigue from baseline to after receiving the intervention. These reports cannot be interpreted as evidence of effectiveness or as a hint of potential therapeutic effects in MS.

All clinical trials assessing the efficacy of an intervention (pharmacologic or non-pharmacologic) on MS fatigue should be randomized, placebo-controlled trials with adequate blinding and allocation concealment. All study procedures, contacts, and visits should be similar between the active intervention and the placebo (sham) group. Being on a waitlist or receiving a control intervention that results in a different intensity of contact with the study personnel is neither adequate nor acceptable.

Allocation concealment might be more feasible for pharmacological interventions, although some medications may result in adverse events that are impossible or unethical to fully mimic in the control group. Allocation concealment is more challenging for rehabilitation, exercise, cognitive behavioral therapy, and diet interventions. Researchers must try to devise a control intervention that mimics the active intervention in all aspects except the essential element(s). For example, in a study to assess the efficacy of cognitive-behavioral therapy, the control group must spend the same amount of time with the same therapists while they withhold the essential elements of the intervention. Similarly, with a dietary intervention, if the active treatment group needs to check and record their glucose levels weekly, the control group should do the same.

The placebo response is an important concept in clinical practice and clinical research. Until we can harness it to improve patient care in an ethical and non-deceptive way (18), it should be carefully considered when designing or evaluating studies that aim to treat MS fatigue.

## Author contributions

MG: Conceptualization, Writing – original draft. EW: Investigation, Supervision, Writing – original draft. BN: Methodology, Writing – original draft, Writing – review & editing.
